# Genome-Wide Association Study of Maize Aboveground Dry Matter Accumulation at Seedling Stage

**DOI:** 10.3389/fgene.2020.571236

**Published:** 2021-01-13

**Authors:** Xianju Lu, Jinglu Wang, Yongjian Wang, Weiliang Wen, Ying Zhang, Jianjun Du, Yanxin Zhao, Xinyu Guo

**Affiliations:** ^1^Beijing Key Laboratory of Digital Plant, Beijing Research Center for Information Technology in Agriculture, National Engineering Research Center for Information Technology in Agriculture, Beijing Academy of Agriculture and Forestry Sciences, Beijing, China; ^2^Beijing Key Laboratory of Maize DNA Fingerprinting and Molecular Breeding, Maize Research Center, Beijing Academy of Agriculture and Forestry Sciences, Beijing, China

**Keywords:** pathways, GWAS, seedling stage, dry matter accumulation, maize

## Abstract

Dry matter accumulation and partitioning during the early phases of development could significantly affect crop growth and productivity. In this study, the aboveground dry matter (DM), the DM of different organs, and partition coefficients of a maize association mapping panel of 412 inbred lines were evaluated at the third and sixth leaf stages (V3 and V6). Further, the properties of these phenotypic traits were analyzed. Genome-wide association studies (GWAS) were conducted on the total aboveground biomass and the DM of different organs. Analysis of GWAS results identified a total of 1,103 unique candidate genes annotated by 678 significant SNPs (*P* value < 1.28e–6). A total of 224 genes annotated by SNPs at the top five of each GWAS method and detected by multiple GWAS methods were regarded as having high reliability. Pathway enrichment analysis was also performed to explore the biological significance and functions of these candidate genes. Several biological pathways related to the regulation of seed growth, gibberellin-mediated signaling pathway, and long-day photoperiodism were enriched. The results of our study could provide new perspectives on breeding high-yielding maize varieties.

## Introduction

Maize (*Zea mays* L.) is an important food staple and feed crop in the world, and the stability of its production is of great significance. Due to continuing population growth and energy insufficiencies, the global demand for food has increased, and thus improvements in maize productivity and quality through breeding are vital (Tester and Langridge, 2010). Plant dry matter is an essential index for evaluating plant growth and development. Dry matter accumulation and partitioning during the early phases of development significantly affect crop growth and yield formation. In maize, dry matter accumulation during the early stages of growth is positively correlated to floret number per ear row (Gonzalez et al., 2019). Therefore, evaluation of the genetic basis of dry matter accumulation and distribution in maize seedlings could explain the genetic mechanism of dry matter accumulation and guide strategies of improving maize yield *via* breeding.

The third and sixth leaf stages (V3 and V6, respectively) are the critical vegetative growth phases. Prior to V3, the seed is the primary nutrient source, and thus seed quality and germination ability affect plant growth, along with the soil temperature and wetness conditions. Once the seedling passes the V3 stage, it transitions from autotrophic to heterotrophic nutrition, and internode elongation begins at the V6 stage. Since the leaf is the primary organ for photosynthesis and transpiration in plants, leaf development affects dry matter accumulation and yield performance (Wang et al., 2011). The maize leaf sheath wraps around the stem and provides strength for the growth and development of the leaf blade (Dong et al., 2019). Prior to V3, the combined strength of leaf sheaths layered on top of one another maintains the upright posture (Lori et al., 2011). Therefore, plant growth and dry matter distribution patterns established at the early stage affect morphogenesis and the photosynthetic competence of the plant at later stages.

Dry matter accumulation is affected by many factors, such as fertilizer, irrigation, and meteorological conditions, among others. However, dry matter accumulation and partitioning in the various organs are quantitative traits controlled by multiple genes. Since the first report on the construction of a genetic linkage map in maize by Helentjaris et al. (1986), many other quantitative trait loci (QTLs) in maize have been identified, including for plant height, ear height, flowering time, and yield-related traits (Wang et al., 2006; Cui et al., 2017). Compared to other crops such as rice and alfalfa, genetic analysis of dry matter-related traits in maize is less reported. In rice, for instance, Li et al. (2008) mapped the QTL controlling dry matter accumulation and partitioning and observed that the traits were controlled by different QTLs at different growth stages. Zhao et al. (2005) identified main-effect QTLs controlling seedling dry matter, seedling height, and 1,000-seed weight. Adhikari et al. (2019) reported QTL associated with spring flowing time and biomass yield of alfalfa. The above studies provide insights into the genetic architecture of dry matter accumulation of maize plants at the seedling stage.

Due to the rapid development of high-density single-nucleotide polymorphism (SNP) assays and associated statistical methods in recent years, genome-wide association studies (GWAS) have become a useful adjunct to classical genetic mapping of quantitative traits in plants (Huang et al., 2010). In maize, GWAS has been successfully used to identify numerous candidate loci/genes controlling various morphological and metabolic traits, such as drought tolerance (Liu et al., 2013; Kang et al., 2015), plant height (Weng et al., 2011; Yang et al., 2014; Wang et al., 2019), ear height (Yang et al., 2014; Farfan et al., 2015), husk traits (Cui et al., 2016), flowering time (Hung et al., 2012; Yang et al., 2014; Li et al., 2016b), mitogen-activated protein kinase cascades (Kong et al., 2013), starch content (Liu et al., 2016), stalk cell wall components (Li et al., 2016a), and many other traits of significant research interest. However, previous studies on the maize plant have focused on the later stages, and GWAS for dry matter accumulation and partition at the seedling stage has been less reported.

The present study examined the total aboveground and organ’s dry matter traits of a maize association mapping panel consisting of 412 inbred lines at V3 and V6 stages. Then, GWAS was conducted to identify the SNPs associated with each phenotypic trait. In total, 1,103 unique candidate genes annotated by 678 significant SNPs (*P* value < 1.28e–6) for dry matter traits were identified. Among these, 224 genes annotated by SNPs that are at the top five of each GWAS method and detected by multiple GWAS methods were regarded as of high reliability. To determine the biological significance and functions of candidate genes, pathway enrichment analysis was also carried out. The differentially expressed genes (DEGs) enriched in various biological pathways related to regulation of seed growth, gibberellin-mediated signaling pathway, and long-day photoperiodism. The results of our study could provide novel targets for breeding high-yielding maize varieties.

## Materials and Methods

### Plant Materials, Growth Conditions, and Sample Collection

In this study, 412 lines that belonged to the maize association mapping panel described by Yang et al. (2011) were used, which were classified into four subgroups based on population structure Q matrix: Stiff stalk (SS) with 27 lines, non-stiff stalk (NSS) with 123 lines, tropical–subtropical (TST) with 165 lines, and admixed group with 97 lines. The plants were planted at the Beijing Academy of Agriculture and Foresting Science, Beijing, China. Maize seeds were planted manually at a depth of 5 cm on 17 May 2019. Each inbred line was planted in two rows of seven plants each. The soil was tilled to a depth of 15 cm before sowing, and the soil texture was loamy sand with a field capacity of 32% in the plow layer. Other chemical properties of the plow layer are as follows: 27.2 g/kg organic matter, 1.34 g/kg total N, 37.6 mg/kg available phosphorus, 91 mg/kg ammonium acetate extractable potassium, and pH 7.6.

Maize growth stages from emergence (VE) to physiological maturity (R6) were recorded. At V3 and V6 stages, three maize plants with uniform growth were sampled by hand and taken to the laboratory to measure dry matter. First, the plants at the V3 stage were divided into leaves and sheaths, while those at the V6 stage were divided into leaves, sheaths, and stalks. Second, the samples were enzymatically deactivated at 105°C for 30 min, oven-dried at 75°C for 72 h, and then weighed (to determine the dry matter). The organ’s dry matter partition coefficient was calculated as the organ’s dry matter divided by the total aboveground dry matter. The dry matter was obtained from the mean of three replicates. Moreover, each phenotypic mean was regarded as the trait and used as the phenotypic data for GWAS.

### ANOVA and Heritability Analysis

Differences between the subpopulation mean of total aboveground dry matter, organ’s dry matter, and organ’s dry matter partition coefficient were assessed by analysis of variance (ANOVA) using the SPSS software version 22.0.

Heritability refers to the percentage of genetic variation (*V*_*A*_) that accounts for the total phenotypic variation, generally denoted by *H*^2^. It can be used to evaluate the correlation between the genetic (σ_*A*_^2^) and environmental (σ_*e*_^2^) factors of a specific phenotypic variation (*V*_*p*_). Heritability (*H*^2^) was calculated for each trait as follows:

$$H^{2}=\frac{V_{A}}{V_{p}}=\frac{\sigma_{A}^{2}}{\sigma_{A}^{2}+\sigma_{e}^{2}}$$

The above analysis was performed in ASReml-R version 4.0 using the “asreml” function of R package *asreml* (Butler, 2009).

### Genome-Wide Association Study

Genotype data were downloaded from the Maizego platform^1^. The SNP data were filtered with a minimum allele frequency (MAF) greater than 0.05 and a call rate greater than 0.01. A total of 779,855 SNPs were retained to conduct the association analysis with phenotypic traits. The population structure was estimated by the STRUCTURE program version 2.3.4 (Hubisz et al., 2009), and the relative kinship was calculated by TASSEL 5 (Bradbury et al., 2007) using 779,855 SNPs. For GWAS, a multi-locus random-SNP-effect mixed linear model tool (R package “mrMLM” version 4.0) (Zhang et al., 2019b) was used on the dry matter phenotypic traits separately to test the statistical association between trait and genotypes separately. Population structure and relative kinship were taken into account in these models. The six ML-GWAS methods (mrMLM, FASTmrMLM, FASTmrEMMA, ISIS EM-BLASSO, pLARmEB, and pKWmEB) were included in the “mrMLM” function. All of these six GWAS methods were done in two steps. In the first step, the *P* value was set as 1.28e–6 (*P* ≤ 1/N, where *N* is the total number of genome-wide SNPs). A default *P* value of 0.0002 was used as the filter threshold for the second step to declare the significance of SNPs associated with a particular trait. The SNP that satisfied all the above methods were regarded as significant SNPs associated with phenotypic traits, and loci that overlapped in multiple methods were considered more reliable. ANNOVAR (Wang et al., 2010), an efficient software tool that utilizes update-to-date information to functionally annotate genetic variants detected from specific genome, was used to complete the annotation of SNPs. All candidate genes were annotated according to the latest maize B73 reference genome (B73 RefGen_v4) available in EnsemblPlants^2^ and NCBI Gene database^3^.

### Functional and Network Analysis

Pathway enrichment analysis was performed by the PlantRegMap database (Jin et al., 2015) and the DAVID online tool (Dennis et al., 2003). The input data consisted of all candidate genes annotated by the significant SNPs associated with dry matter phenotypic traits. Gene Ontology (GO) (Ashburner et al., 2000) terms and Kyoto Encyclopedia of Genes and Genomes (KEGG) pathways (Kanehisa, 2002) with *P* value < 0.05 were regarded significant.

The Cytoscape v3.7.2 open-source software platform (Shannon et al., 2003) was used to visualize the complex trait–gene network and integrate the input data by their attribute information.

## Results

### Phenotypic Variations of Measured Quantitative Traits

The dry matter accumulation and partition traits followed a normal distribution (Supplementary Figures 1, 2). Extensive phenotypic variations were observed for dry matter traits at V3 and V6 stages in this maize panel, as shown by the descriptive statistics in Table 1. The total dry matter and organ’s dry matter at V3 and V6 had a higher phenotypic variation, with the variable coefficients ranging from 0.43 to 0.55. Meanwhile, the partition coefficients of dry matter at V3 and V6 had a lower variable coefficient, ranging from 0.04 to 0.27.

**TABLE 1:** Descriptive statistical analysis of dry matter-related traits in this study^1)^.

**Traits**	**Growth stage**	**Organ^2)^**	**Min (g)**	**Max (g)**	**Mean ± SD (g)**	**CV**
Dry matter	V3	Total DM	0.0367	0.3233	0.1142 ± 0.0495	0.43
		Leaf DM	0.0200	0.1933	0.0603 ± 0.0296	0.49
		Sheath DM	0.0117	0.1600	0.0539 ± 0.0261	0.48
	V6	Total DM	0.42	8.33	3.15 ± 1.36	0.43
		Leaf DM	0.31	6.32	2.45 ± 1.06	0.43
		Sheath DM	0.07	1.62	0.54 ± 0.25	0.46
		Stalk DM	0.01	0.58	0.16 ± 0.08	0.55
Partition coefficient	V3	Leaf PC	0.21	0.84	0.53 ± 0.11	0.2
		Sheath PC	0.16	0.78	0.47 ± 0.11	0.23
	V6	Leaf PC	0.63	0.87	0.78 ± 0.03	0.04
		Sheath PC	0.09	0.32	0.17 ± 0.03	0.17
		Stalk PC	0.01	0.09	0.05 ± 0.01	0.27

*^1)^SD, standard deviation; CV, coefficient of variation. ^2)^DM, dry matter; PC, partition coefficient, as the ratio of organ’s dry matter to total aboveground dry matter.*

**FIGURE 1:**
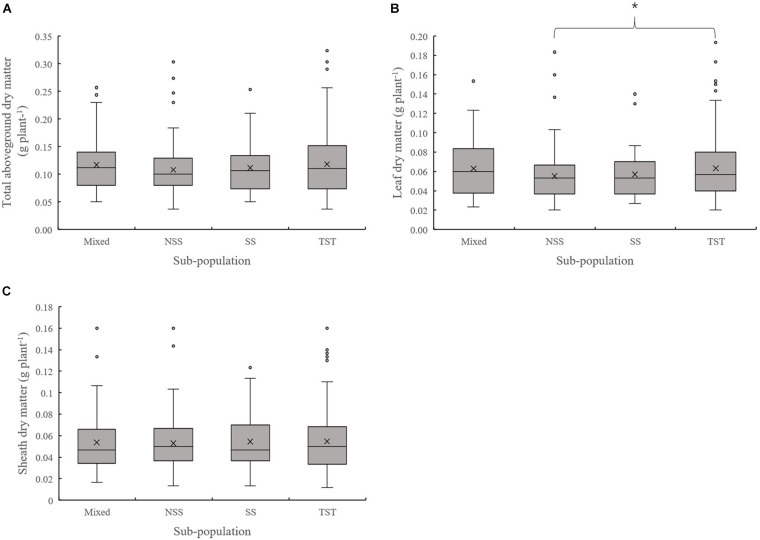
Boxplot of the total aboveground dry matter and organ’s dry matter of different subpopulations at the V3 stage. * denotes significant differences between subpopulations at *P* ≤ 0.05. **(A–C)** represent the Total aboveground dry matter, Leaf dry matter and Sheath dry matter of different subpopulations respectively.

**FIGURE 2:**
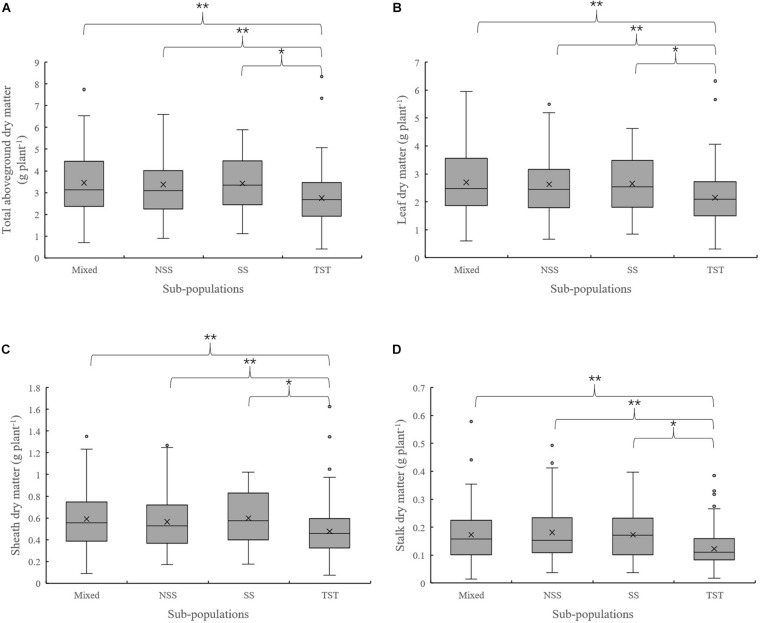
Boxplot of the total aboveground dry matter and organ’s dry matter in different subpopulations at the V6 stage. ** denotes significant differences between subpopulations at *P* ≤ 0.01, * denotes significant differences between subpopulations at *P* ≤ 0.05. **(A–D)** represent total aboveground dry matter, Leaf dry matter, Sheath dry matter and Stalk dry matter in different subpopulations respectively.

The results of population structure and kinship analysis were in line with a previous study (Yang et al., 2011), and the proposed classification of population structure was learned from our study and also as a basis for further analysis of phenotypic data. Based on the dry matter phenotypic indicators and the organ’s dry matter partition coefficients at V3 and V6, an ANOVA was done to evaluate for differences among the four subpopulations in the panel. For dry matter accumulation at V3, the leaf dry matter in NSS was significantly lower than the TST subpopulation. There were no significant differences in total dry matter and sheath dry matter among subpopulations (Figure 1). At V6, the total aboveground, leaf, sheath, and stalk dry matter of Mixed, NSS, and SS subpopulations were significantly higher than in the TST subpopulation, suggesting that maize inbred lines from tropical or subtropical origin tend to grow slower. The differences among the Mixed, NSS, and SS subpopulation for total aboveground, leaf, sheath, and stalk dry matter were not significant (Figure 2).

**FIGURE 3:**
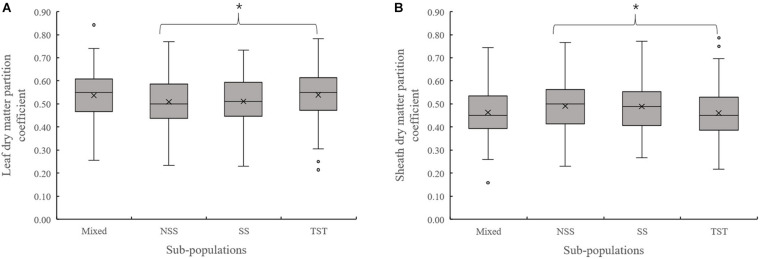
Boxplot of the different organ’s dry matter partition coefficients in different subpopulations at the V3 stage. * denotes significant differences between subpopulations at *P* ≤ 0.05. **(A)** Leaf dry matter partition coefficient. **(B)** Sheath dry matter partition coefficient.

**FIGURE 4:**
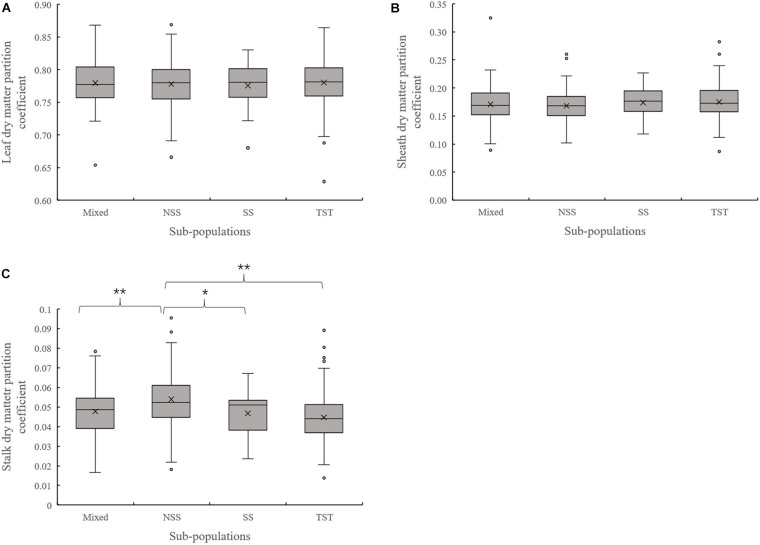
Boxplot of the different organ’s dry matter partition coefficients in different subpopulations at the V6 stage. ** denotes significant differences between subpopulations at *P* ≤ 0.01. * denotes significant differences between subpopulations at *P* ≤ 0.05. **(A)** Leaf dry matter partition coefficient. **(B)** Sheath dry matter partition coefficient. **(C)** Stalk dry matter partition coefficient.

Evaluation of the organ’s dry matter partition coefficient at V3 showed that the leaf dry matter partition coefficient in NSS subpopulation was significantly lower than that in the TST subpopulation. Meanwhile, the sheath dry matter partition coefficient in the NSS subpopulation was substantially higher than that in the TST subpopulation (Figure 3). At V6, there were no significant differences in leaf and sheath dry matter partition coefficient among the four subpopulations. However, the stalk dry matter partition coefficient of NSS was significantly higher than in the Mixed, SS, and TST subpopulations. There were no significant differences for stalk dry matter partition coefficient among the Mixed, SS, and TST subpopulation (Figure 4).

### Heritability Analysis

**FIGURE 5:**
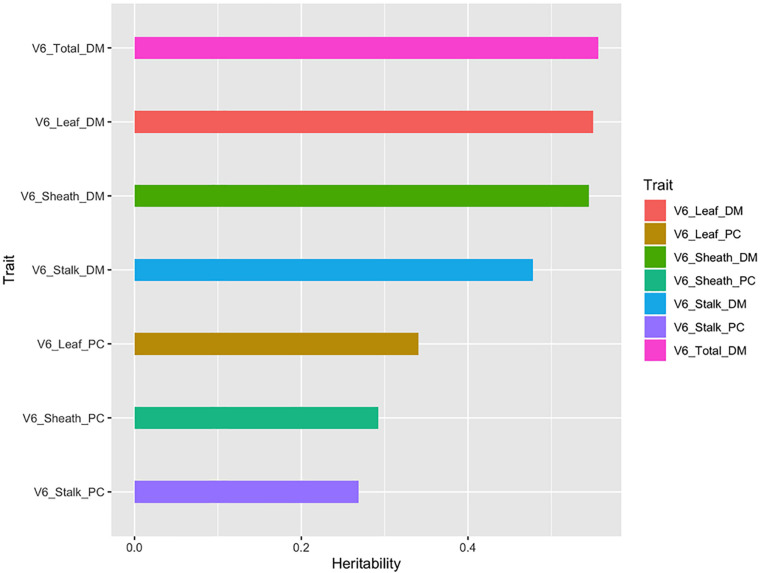
The broad-sense heritability (*H*^2^) of dry matter related traits at the V6 stage.

The heritability of a trait is one of the key parameters considered in designing and selecting plant breeding schemes (Chen and Lübberstedt, 2010; Holland et al., 2010). As shown in Figure 5, the total aboveground dry matter, the dry matter of the different organs (leaf, sheath, and stalk), and the partition coefficient showed different heritability patterns, ranging from 0.261 to 0.591. The total aboveground dry matter and organ’s dry matter had heritability coefficients greater than 0.4, while the heritability of the partition coefficients of different organs was less than 0.4. It could be clearly seen that the variability in organ’s dry matter was greatly affected by genetic factors. However, the partition coefficient calculated from this set of data in our study did not show that it is largely affected by genetic factors. Therefore, according to the heritability values, three traits (total aboveground dry matter, leaf dry matter, and sheath dry matter) at V3 and four traits (total aboveground dry matter, leaf dry matter, sheath dry matter, and stalk dry matter) at V6 were used for subpopulation variation analysis and SNP identification.

### Significant SNPs Obtained by GWAS

This study used multi-locus random-SNP-effect mixed linear models in the R package “mrMLM” (version 4.0) for genome-wide association analysis of dry matter accumulation and partition traits. The model identified a total of 678 significant SNPs (*P* value < 1.28e–6) associated with target traits. Because these results were a collection of six GWAS methods, the top five most significant SNPs obtained by each method and the SNPs validated by two or more methods were considered as highly significant results. Thus, the reliability of these results could be higher than that of the others. Consequently, 129 highly significant associated SNPs were filtered for all dry matter traits. Among them, 22, 14, and 20 highly significant SNPs were identified for the leaf dry matter, sheath dry matter, and total aboveground dry matter of V3, respectively. Additionally, 27, 22, 21, and 25 highly significant SNPs were detected for the leaf dry matter, sheath dry matter, stalk dry matter, and total aboveground dry matter of V6, respectively. The detailed statistical results are shown in Table 2.

**TABLE 2:** Statistics and summary of significant loci obtained by GWAS for each trait.

**Trait^1)^**	**No. of significant SNPs**	**Gene no. of significant SNPs located**	**No. of significant SNPs listed in the top 5 and validated by multiple methods**	**Gene no. of significant SNPs listed in the top 5 and validated by multiple methods**	**Chromosomes of SNPs listed in the top 5 and validated by multiple methods**
V3 Leaf DM	289	492	22	32	1, 3, 5, 7, 8, 9, 10
V3 Sheath DM	230	384	14	25	1, 2, 3, 4, 5, 7, 8, 9
V3 Total DM	32	57	20	38	1, 2, 3, 4, 5, 6, 7, 8, 10
V6 Leaf DM	59	105	27	49	1, 2, 3, 4, 5, 7, 8, 10
V6 Sheath DM	34	60	22	39	1, 2, 3, 4, 5, 6, 7, 9, 10
V6 Stalk DM	41	70	21	35	1, 2, 3, 4, 5, 8, 9
V6 Total DM	47	85	25	44	1, 2, 3, 4, 5, 6, 7, 8, 9, 10
Summary	678	1,103	129	224	

*^1)^V3 Leaf DM, V3 Sheath DM and V3 Total DM: the dry matter of leaf, sheath and total aboveground dry matter at V3 stage; V6 Leaf DM, V6 Sheath DM, V6 Stalk DM, and V6 Total DM: the dry matter of leaf, sheath, stalk, and total aboveground dry matter at V6 stage.*

### Identification and Annotation of Candidate Genes

All candidate genes were annotated according to the latest maize B73 reference genome (B73 RefGen_v4) available in EnsemblPlants and NCBI Gene databases. In total, 1,103 unique candidate genes were annotated by the 678 significant associated SNPs. The number of genes annotated by SNPs listed in both the top five of the results of each method and results validated by multiple methods was 224 (Table 2). Among them, the numbers of genes associated with each V3 trait were 32 (V3 Leaf DM), 25 (V3 Sheath DM), and 38 (V3 Total DM). Besides, at V6, the corresponding gene numbers were 49 (V6 Leaf DM), 39 (V6 Sheath DM), 35 (V6 Stalk DM), and 44 (V6 Total). It could be seen that dry matter traits did not have overlapping candidate genes. Subsequently, the 224 highly significant candidate genes were further annotated using the NCBI database, after which 158 genes with detailed functional descriptions were obtained (Supplementary Table 1). The 158 genes were found to be distributed in 10 chromosomes. Detailed information of each gene and its related trait is presented in Supplementary Table 1. Out of these, 18 genes (*GRMZM2G105571*, *Zm00001d027469*, *Zm00001d043622*, *GRMZM2G353553*, *GRMZM2G167856*, *GRMZM2G171373*, *GRMZM2G092120*, *CKX10*, *GRMZM2G035217*, *GRMZM2G128644*, *GRMZM2G314064*, *GRMZM2G407825*, *GRMZM6G865522*, *GRMZM2G045732*, *pco092737*, *Zm00001d002111*, *GRMZM2G042412*, and *GRMZM2G061537*) were associated with a minimum of two traits. In addition, some of them [*GRMZM2G105571* (Li et al., 2018), *GRMZM2G167856* (Li et al., 2016), *GRMZM2G171373* (Aguilera-Alvarado et al., 2019), *CKX10* (Smehilova et al., 2009), *GRMZM2G128644* (Song et al., 2016), *GRMZM2G314064* (Jiang et al., 2012), *GRMZM2G407825* (Yan et al., 2017), etc.] had been reported by previous studies of maize seed and leaf, which was consistent with our study. In terms of this, nearly half of the genes obtained in this study were proofed by previous researches, indicating the reliability of our results. Therefore, the new genes identified by our analysis process could also become a powerful reference for peer research.

An additional 57 genes identified by multiple GWAS methods are shown in Supplementary Table 1 (marked as “Y”). Since these genes were annotated by the top five SNPs of each methods and then validated by multiple methods, their reliability is considered higher than that of other genes. Among these 57 genes, 43 were only related to one phenotypic trait. For V3, *GRMZM2G340279*, *GRMZM2G347808*, *GRMZM2G018782*, *GRMZM2G099678*, *GRMZM2G153127*, *Zm00001d042998*, *GRMZM2G064437*, and *GRMZM2G030284* were associated with leaf dry matter. The candidate genes of sheath dry matter were *GRMZM2G147917*, *GRMZM2G164088*, *GRMZM2G138770*, *GRMZM2G032163*, *GRMZM2G077004*, *GRMZM2G092616*, *GRMZM5G884544*, *GRMZM2G004119*, and *GRMZM2G152815*. For V6, only two genes (*Zm00001d043484* and *GRMZM2G074689*) were associated with leaf dry matter, while *GRMZM2G086707*, *GRMZM2G148706*, *GRMZM2G448701*, *GRMZM2G166718*, *GRMZM2G166776*, *GRMZM2G016393*, *Zm00001d020588*, and *Zm00001d020589* were related to sheath dry matter. Meanwhile, the V6 candidate genes of stalk dry matter were *GRMZM2G107838*, *GRMZM2G050845*, *GRMZM2G085509*, *GRMZM2G160136*, *GRMZM2G397759*, *GRMZM2G026892*, and *GRMZM2G060349*.

### Pathways Enriched by Functional Enrichment Analysis

**FIGURE 6:**
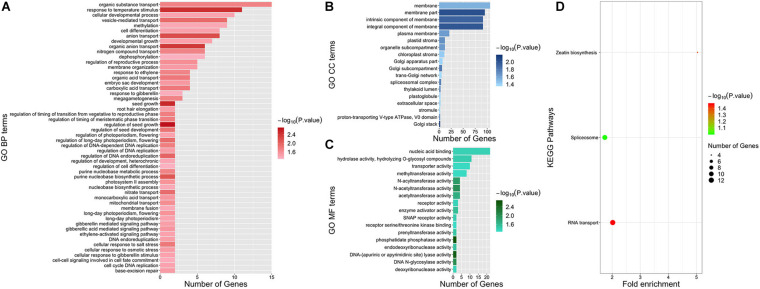
Functional enrichment results of all candidate genes. Panels **(A–C)** represent GO BP, GO CC, and GO MF terms, respectively. GO terms are shown on the left. Different colors indicate the significance of GO terms. The length of each bar represents the gene numbers enriched in each term. Panel **(D)** illustrates KEGG pathways. Pathways are shown on the left. Different colors indicate the significance of pathways. The size of the dot represents the number of genes enriched in each pathway.

Functional enrichment analysis was conducted to further explore the function of genes associated with dry matter traits. After uploading the candidate gene IDs of all dry matter traits to PlantRegMap and DAVID, a total of 87 GO terms and two KEGG pathways (*P* value < 0.05) were enriched (Figure 6). Among them, 52 terms were GO BP (Biological Process) terms. Two GO BP terms “regulation of seed growth” (GO:0080113, *P* value = 0.0021) and “seed growth” (GO:0080112, *P* value = 0.003) both contained genes *GRMZM2G035156* and *GRMZM2G042101* and were obtained with the highest significance. Strikingly, several gibberellin-related terms enriched by *GRMZM2G120320* and *GRMZM2G348238* were obtained with a high significance (*P* value < 0.05), such as “gibberellic acid mediated signaling pathway” (GO:0009740), “gibberellin mediated signaling pathway” (GO:0010476), “cellular response to gibberellin stimulus” (GO:0071370), and “response to gibberellin” (GO:0009739). Besides, the effect of light on dry matter accumulation was found remarkable. Notably, four GO BP terms enriched by *GRMZM2G139038* and *GRMZM2G348238* were related to photoperiodism, and three of them were about long-day photoperiodism. These terms were “regulation of long-day photoperiodism, flowering” (GO:0048586), “long-day photoperiodism, flowering” (GO:0048574), “long-day photoperiodism” (GO:0048571), and “regulation of photoperiodism, flowering” (GO:2000028). In addition, two interesting GO BP terms involved in the regulation of timing of phase transition were obtained, namely, “regulation of timing of meristematic phase transition” (GO:0048506) and “regulation of timing of the transition from vegetative to reproductive phase” (GO:0048510). In addition, the two significant KEGG pathways “RNA transport” (zma03013, *P* value = 0.0341) and “Zeatin biosynthesis” (zma00908, *P* value = 0.0434) were enriched by DAVID (Figure 6D).

### Trait–Gene Network Visualization

**FIGURE 7:**
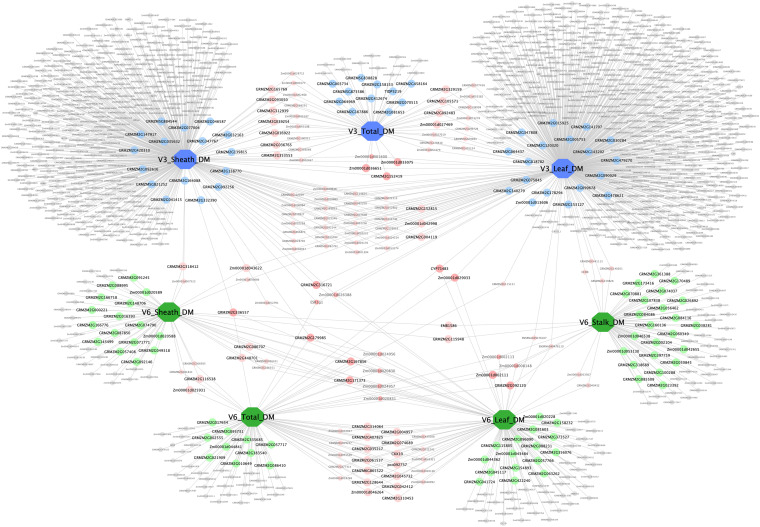
Trait–gene network of dry matter traits. The trait–gene network constructed by dry matter traits and their related genes. Traits and genes are shown in different shapes and sizes. Of the seven large octagon nodes, the three blue nodes represent stage V3 traits (V3 Leaf DM, V3 Sheath DM, and V3 Total DM), and the four green nodes represent stage V6 traits (V6 Leaf DM, V6 Sheath DM, V6 Stalk DM, and V6 Total DM). Genes are represented by round nodes, and different colors and size indicate different attributes. The 158 genes with detailed functional descriptions were labeled as large round nodes, with colors corresponding to their related traits. The gray small nodes represent genes that correlate with only one specific trait, and the pink and red nodes (both large and small ones) indicate two-trait shared genes and three-trait shared genes, respectively.

A complex network consisting of dry matter traits and their candidate genes was constructed by Cytoscape v3.7.2 (Figure 7). The network contained 7 large nodes (phenotypic traits) and 1,103 small round nodes (candidate genes), and 1,253 edges (the interactions between traits and genes). V3 and V6 traits were marked blue and green, respectively, to make a distinction between two vegetative stages. Candidate genes with different colors represented the diversity of interactions. Among these, the 158 genes with detailed functional descriptions were labeled as large round nodes, with colors corresponding to their related traits. The other genes were shown as small round nodes, and the colors indicated different meaning. The gray nodes represented genes that correlate with one specific trait, and the pink and red ones indicated two-trait shared genes and three-trait shared genes, respectively. It was apparent that many genes were shared between traits at the same stage, suggesting that the growth and development of various organs at the same stage was a collaborative process. However, there were also 19 shared genes among traits at different stages, with the possibility of regulating the aboveground dry matter accumulation process of maize during the entire seedling stage.

## Discussion

Maize is originated from tropical regions and subsequently adapted into subtropical regions, so population structure may have imposed effects on maize growth and development (Camus-Kulandaivelu et al., 2006). Several research achievements employing the maize association panel used in this study have been reported. These studies include grain regulation network (Jin et al., 2016; Liu et al., 2016), flowering date (Yang et al., 2013, 2014), husk traits (Cui et al., 2016), forage quality (Wang et al., 2016), yield-related traits (Yang et al., 2014; Liu et al., 2015), disease resistance (Chen et al., 2015; Ding et al., 2015), and drought tolerance (Liu et al., 2013; Mao et al., 2015; Zhang et al., 2016), among others. However, genetic analysis of dry matter accumulation for this association panel has been less reported. According to the information of population structure in a previous study (Yang et al., 2011), phenotypic variation of dry matter accumulation and partition among subpopulations was compared and differences were identified (Figures 1–4). In this study, the difference between subpopulations was not significant, except that the leaf dry matter in TST was higher than that in NSS at the V3 stage. However, the total aboveground dry matter and different organs’ dry matter in NSS, SS, and Mixed subpopulations were significantly higher than that of the TST subpopulation at V6. The results of this study suggest that genotype differences in dry matter accumulation may not be substantially expressed at the V3 stage, and genetic effects increase with the development process. Also, the results of this study indicate that TST maize grew slower than NSS, SS, and Mixed subpopulations at the seedling stage. Wang et al. (2019) also reported that TST maize grows slower and is shorter than TEM (temperate lines) maize, from sowing to the V12 stage. Photoperiod is an important factor affecting plant development. The tropical–subtropical line is sensitive to photoperiod. The longer sunshine in temperate regions may be the reason that leads to slower growth of tropical–subtropical lines. In addition, several GO terms including photoperiodism were enriched by the candidate genes related to the traits in our study.

Proper plant growth and development during the early stages is of significance, as it establishes the normal plant structure at later stages. Besides, it ensures that the plant can carry out all physiological and metabolic processes to obtain maximal yield. Previous research has shown that seed germination and plant development are affected by plant hormones, such as gibberellin and abscisic acid, and environmental factors such as light, water, and temperature. In this study, functional enrichment of all candidate genes related to dry matter traits identified several biological pathways related to seed growth and development, gibberellin, photoperiodism, and temperature. In total, three GO BP terms were enriched, “regulation of seed growth” (GO:0080113, *P* value = 0.0021), “seed growth” (GO:0080112, *P* value = 0.003), and “regulation of seed development” (GO:0080050, *P* value = 0.0139) containing *GRMZM2G035156* and *GRMZM2G042101*. *GRMZM2G035156* and *GRMZM2G042101* were detected as candidate genes for V6 Leaf DM and V3 Sheath DM, respectively. These findings indicate that genes related to seed growth and development are involved in the growth and development of multiple organs during the maize seedling stage. Additionally, several gibberellin-related GO terms were enriched by *GRMZM2G120320* and *GRMZM2G348238*. Gibberellin is a key plant hormone that regulates the developmental switch between seed dormancy and germination, juvenile and adult growth phases, and vegetative and reproductive development. Gibberellins occur in seeds, young leaves, and roots. It has been reported that gibberellin/abscisic acid balance could govern germination versus maturation pathways in maize (White et al., 2000). In our study, *GRMZM2G120320* was found in GO terms “gibberellic acid-mediated signaling pathway” (GO:0009740), “gibberellin mediated signaling pathway” (GO:0010476), “cellular response to gibberellin stimulus” (GO:0071370), and “response to gibberellin” (GO:0009739). This result suggests that *GRMZM2G120320* is involved in gibberellin-related pathways and could play various roles in plant growth, organ development, and response to environmental stimuli. *GRMZM2G120320* is a gene of the WRKY transcription factor family. Accumulating evidence suggests that WRKY proteins play significant roles in response to biotic and abiotic stresses and in development (Eulgem et al., 2000; Ulker and Somssich, 2004; Zhang and Wang, 2005). *GRMZM2G120320* has been reported in a study of root phenotype and dynamic transcriptome analysis in maize and was found to be mainly associated with drought response by the primary root (Zhang et al., 2019a). Notably, the present study identified *GRMZM2G120320* as a candidate gene regulating V3 leaf dry matter and was annotated by the top five SNPs listed in our results. Hence, we infer that this gene is involved in the growth and development of the maize leaf and has an influence on the accumulation of leaf dry matter during the V3 stage. The gene, *GRMZM2G348238*, is also a candidate for V3 leaf DM. In addition to the gibberellin-related pathways, *GRMZM2G348238* also appeared in long-day photoperiodism and temperature-related pathways. Although the specific function of this gene is obscure, these results suggest that *GRMZM2G348238* is sensitive to environmental changes, such as light and temperature, and participates in leaf growth and dry matter accumulation at the V3 stage.

*GRMZM2G332390* [16-auxin-responsive SAUR (small auxin up-regulated RNA) family member] was annotated by the top five SNPs listed in the GWAS results and was identified as a candidate gene of V3 sheath DM. Interestingly, the gene was also detected in a genome expression profile analysis of the maize sheath and is involved in the resistance of maize plants to *Rhizoctonia solani* infection (Gao et al., 2014). This report by Gao et al. (2014) is consistent with our GWAS results that *GRMZM2G332390* controls sheath-related traits. Based on this result, we infer that *GRMZM2G332390* is a credible candidate gene of sheath dry matter during the V3 stage.

For the V6 stage, *CKX10* (cytokinin dehydrogenase 10) was found as a candidate gene of leaf dry matter with high reliability, for it was not only annotated by the top five SNPs of each GWAS method but also validated by multiple methods. *CKX10* is a member of the *CKX* gene family. Plenty of research work on this gene family has been done in Poaceae (Mameaux et al., 2012), including several studies in maize. In maize leaves, for instance, the expression of *CKX10* in young leaves is high but markedly downregulated in senescent tissues (Smehilova et al., 2009). Also, a transcriptome analysis of maize identified *CKX10* as one of the DEGs related to hormone metabolism (Zheng et al., 2020). It can be seen that *CKX10* is a gene related to the growth and development of maize leaves. In this study, it is exactly identified as a candidate gene of leaf dry matter. Therefore, we could infer that *CKX10* plays a key role in maize leaves at seedling stage.

The number of studies on the topic of maize GWAS has increased year by year, and the number of articles published in the past 5 years has increased dramatically. It shows that GWAS has been widely used in the genetic research of maize and has gradually become an important means to explore the genetic mechanism of maize agronomic traits and key phenotypes (Xiao et al., 2016; Zhang et al., 2016; Zhou et al., 2016; Dai et al., 2018; Owens et al., 2019). Yang et al. (2014) conducted GWAS on 17 agronomic traits in 513 maize inbred lines with 500,000 high-quality SNP markers by using mixed linear model and a new method [Anderson–Darling (A–D) test]. The trait-related SNPs and candidate genes obtained in this study provided abundant resources for maize genetic breeding. In the future, researchers can utilize QTL, qRT-PCR, and other methods to verify the SNPs and candidate genes identified by GWAS, which can enhance the reliability of research results and enrich the findings of maize genetics. In addition, the combination of phenomics and GWAS will break the existing phenotypic bottleneck and greatly advance the genetic research process of maize.

Our study provides a credible list of candidate genes related to dry matter traits in maize seedlings for peer research. Additionally, multiple methods were used to conduct GWAS. On the one hand, it could make up for the deficiencies between various methods and help us obtain abundant genetic loci. On the other hand, it could increase the credibility of the results that have been identified by multiple methods. The present study mainly focused on the weight of maize dry matter at the seedling stage. Our next study will explore other phenotypic traits such as leaf morphology and plant 3D morphology at the seedling stage and evaluate the genetic mechanisms of maize growth and development at an early stage.

## Data Availability Statement

Genotypic data that support the findings of this research are open resource and can be downloaded from http://www.maizego.org/. All other data are available from corresponding author upon reasonable request.

## Author Contributions

XL and JW drafted and revised the manuscript. XG proposed the conceptualization of this study and reviewed the manuscript. YW, WW, YZ, JD, and YZ performed field experiments and obtained phenotypic data. XL and JW analyzed and interpreted the results. All authors contributed to the article and approved the submitted version.

## Conflict of Interest

The authors declare that the research was conducted in the absence of any commercial or financial relationships that could be construed as a potential conflict of interest.

## Footnotes

1 www.maizego.org/Resources.html

2 http://plants.ensembl.org/Zea_mays/Info/Index

3 https://www.ncbi.nlm.nih.gov/gene

## Supplementary Material

Supplementary Figure 1 Frequency distribution of the total aboveground dry matter and organ’s dry matter at the V3 and V6 stage. **(A–C)** represent the results at V3 stage and **(D–G)** represent the results at V6 stage.

Supplementary Figure 2 Frequency distribution of the organ’s dry matter partition coefficient at the V3 and V6 stage. **(A,B)** represent the results at V3 stage and **(C–E)** represent the results at V6 stage.

Supplementary Table 1 Gene annotation of GWAS significant loci.
