# Supplementary figures and images for: Genome-Wide Association Study of Maize Aboveground Dry Matter Accumulation at Seedling Stage
Source: Front Genet. 2021 Jan 13;11:571236. doi: 10.3389/fgene.2020.571236 (PMC7838602; doi:10.3389/fgene.2020.571236)

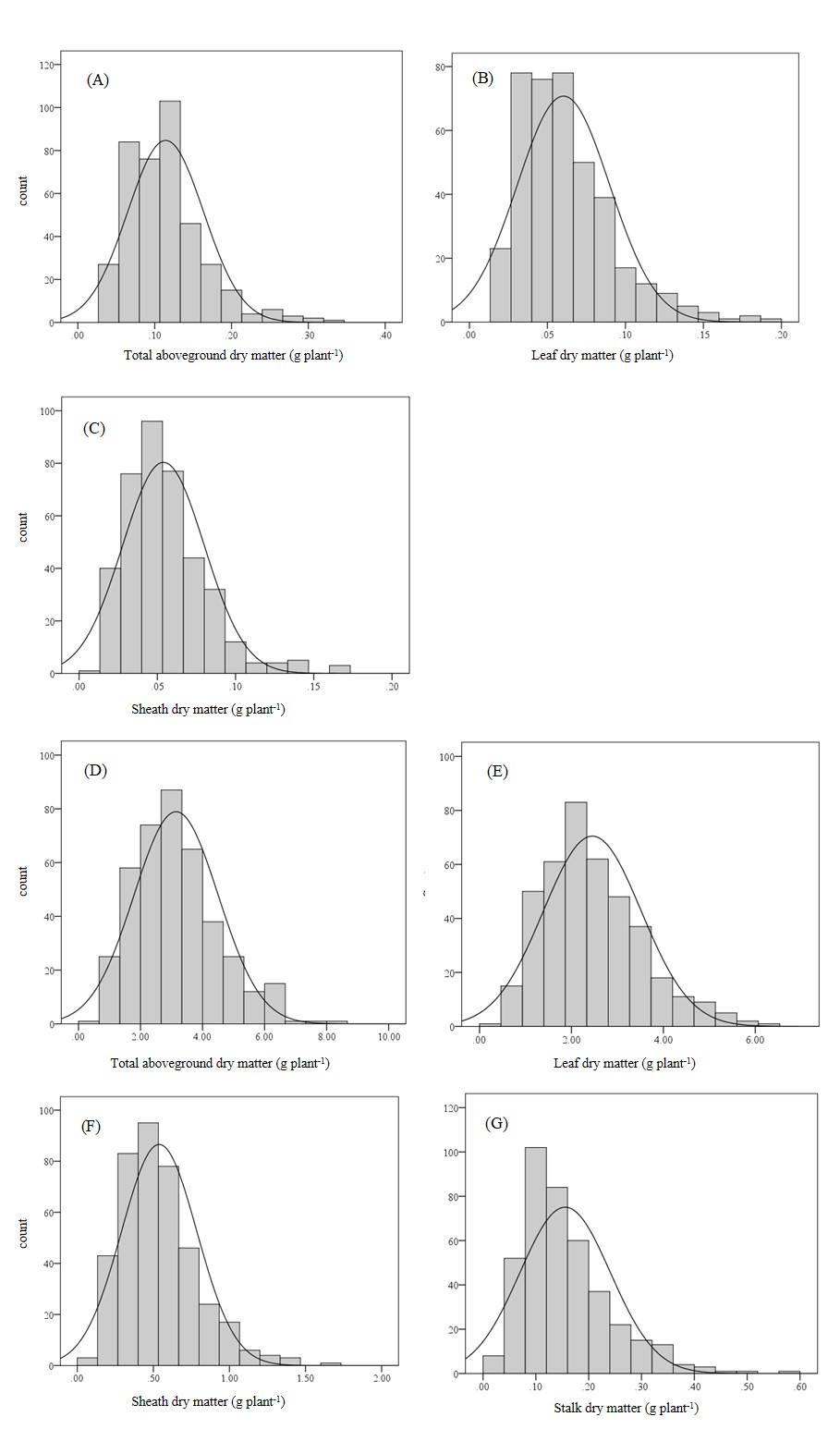

Supplement: Supplementary Figure 1 — Frequency distribution of the total aboveground dry matter and organ’s dry matter at the V3 and V6 stage. (A–C) represent the results at V3 stage and (D–G) represent the results at V6 stage. [file Image_1.JPEG]

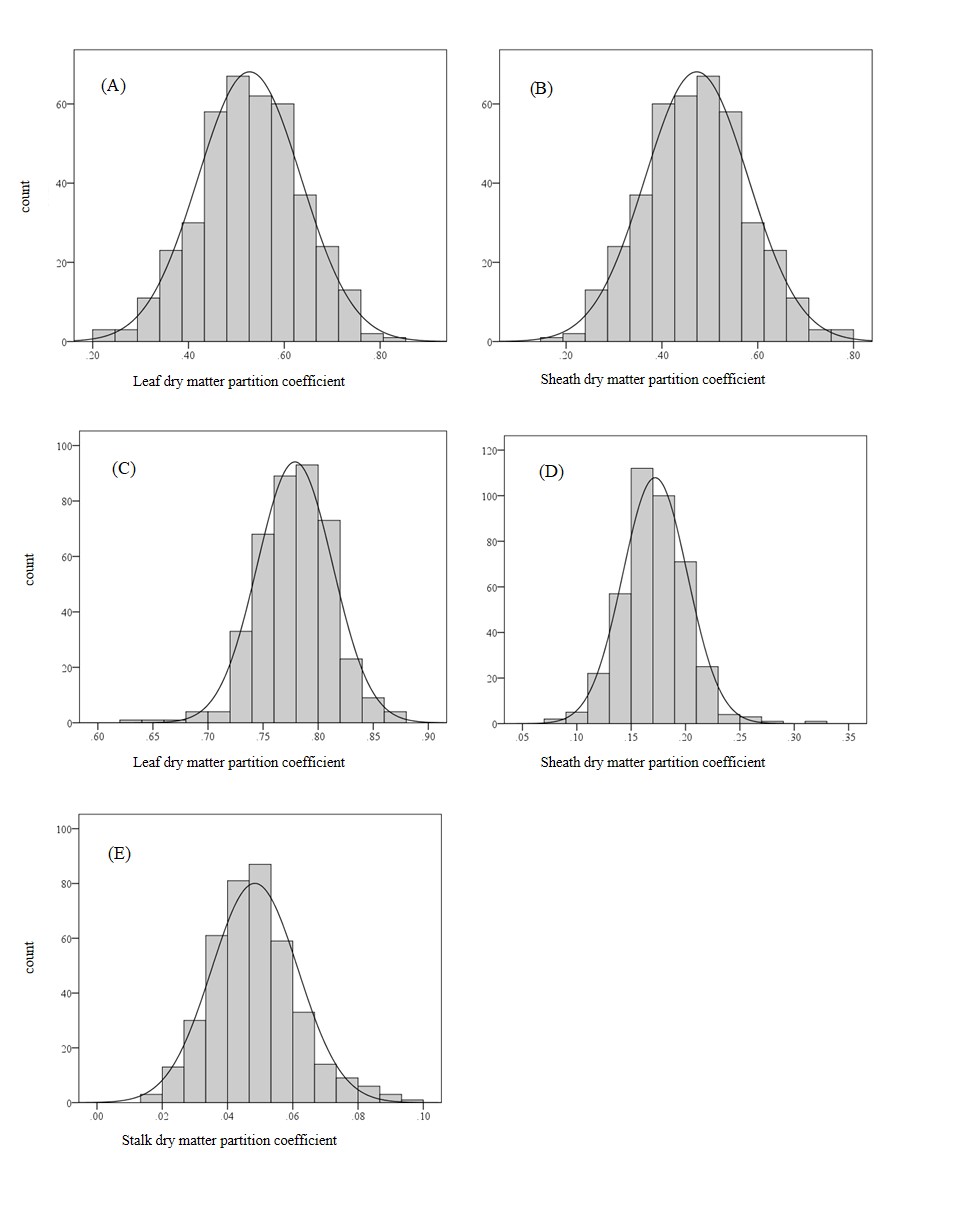

Supplement: Supplementary Figure 2 — Frequency distribution of the organ’s dry matter partition coefficient at the V3 and V6 stage. (A,B) represent the results at V3 stage and (C–E) represent the results at V6 stage. [file Image_2.JPEG]
