# Supplementary material for: Genome-Wide Association Study of Maize Aboveground Dry Matter Accumulation at Seedling Stage
Source: Front Genet. 2021 Jan 13;11:571236. doi: 10.3389/fgene.2020.571236 (PMC7838602; doi:10.3389/fgene.2020.571236)
Supplement: Supplementary Table 1 — Gene annotation of GWAS significant loci. [file Table_1.DOCX]

**Supplementary Table S1** Gene annotation of GWAS significant loci

| **Gene** | **Description** | **Chromosome** | **Genomic_nucleotide_accession.version** | **Start_position_on_the_genomic_accession** | **End_position_on_the_genomic_accession** | **SNP** | **Positional relationship between SNP and gene** | **Related trait** | **Validated_by_multiple_methods** |
| --- | --- | --- | --- | --- | --- | --- | --- | --- | --- |
| GRMZM2G005753 | Chaperone protein dnaJ 15 | 3 | NC_024461.2 | 130023659 | 130034790 | chr3.S_129970931 | intergenic | V3 Leaf DM |  |
| GRMZM2G015925 | Polynucleotidyl transferase ribonuclease H-like superfamily protein | 1 | NC_024459.2 | 223125139 | 223133039 | chr1.S_223113451 | intergenic | V3 Leaf DM |  |
| GRMZM2G018782 | probable mediator of RNA polymerase II transcription subunit 26b | 3 | NC_024461.2 | 47375333 | 47386683 | chr3.S_47398640 | intergenic | V3 Leaf DM | Y |
| GRMZM2G030284 | uncharacterized LOC100191450 | 8 | NC_024466.2 | 156039720 | 156043888 | chr8.S_155978615 | intergenic | V3 Leaf DM | Y |
| GRMZM2G064437 | proton myo-inositol cotransporter | 7 | NC_024465.2 | 166550980 | 166552956 | chr7.S_166564979 | intergenic | V3 Leaf DM | Y |
| GRMZM2G075845 | uncharacterized LOC100275772 | 5 | NC_024463.2 | 95406964 | 95408416 | chr5.S_95233997 | intergenic | V3 Leaf DM |  |
| GRMZM2G090029 | Cyclin-dependent protein kinase inhibitor SMR1 | 1 | NC_024459.2 | 88467708 | 88468805 | chr1.S_88482271 | intergenic | V3 Leaf DM |  |
| GRMZM2G099678 | uncharacterized LOC100282200 | 3 | NC_024461.2 | 1325192 | 1327614 | chr3.S_1353982 | intergenic | V3 Leaf DM | Y |
| GRMZM2G120320 | uncharacterized LOC100279570 | 5 | NC_024463.2 | 95183903 | 95185602 | chr5.S_95233997 | intergenic | V3 Leaf DM |  |
| GRMZM2G141707 | uncharacterized LOC100282002 | 1 | NC_024459.2 | 223035489 | 223037373 | chr1.S_223113451 | intergenic | V3 Leaf DM |  |
| GRMZM2G143202 | carotene epsilon-monooxygenase, chloroplastic | 1 | NC_024459.2 | 88693293 | 88704195 | chr1.S_88482271 | intergenic | V3 Leaf DM |  |
| GRMZM2G153127 | putative E3 ubiquitin-protein ligase LIN | 3 | NC_024461.2 | 1385311 | 1392159 | chr3.S_1353982 | intergenic | V3 Leaf DM | Y |
| GRMZM2G178294 | uncharacterized LOC100304428 | 9 | NC_024467.2 | 150475829 | 150486465 | chr9.S_150487000 | upstream;downstream | V3 Leaf DM |  |
| GRMZM2G340279 | pentatricopeptide repeat-containing protein At4g33990 | 1 | NC_024459.2 | 70124254 | 70126690 | chr1.S_70163708 | intergenic | V3 Leaf DM | Y |
| GRMZM2G347808 | uncharacterized LOC100277845 | 1 | NC_024459.2 | 70211439 | 70219076 | chr1.S_70163708 | intergenic | V3 Leaf DM | Y |
| GRMZM2G478621 | AP-3 complex subunit delta | 3 | NC_024461.2 | 129952002 | 129955962 | chr3.S_129970931 | intergenic | V3 Leaf DM |  |
| GRMZM2G479270 | WEB family protein At1g12150 | 9 | NC_024467.2 | 150487588 | 150489897 | chr9.S_150487000 | upstream;downstream | V3 Leaf DM |  |
| Zm00001d013606 | serine-threonine protein kinase pseudogene | 5 | NC_024463.2 | 15246164 | 15252271 | chr5.S_15186830 | intergenic | V3 Leaf DM |  |
| Zm00001d042998 | uncharacterized LOC100382623 | 3 | NC_024461.2 | 186202012 | 186204465 | chr3.S_186210301 | intergenic | V3 Leaf DM | Y |
| GRMZM2G105571 | uncharacterized LOC100284305 | 1 | NC_024459.2 | 5943755 | 5947506 | chr1.S_5995473 | intergenic | V3 Leaf DM,V3 Total DM |  |
| Zm00001d027469 | cytochrome c oxidase assembly protein COX19 | 1 | NC_024459.2 | 6020751 | 6023314 | chr1.S_6016071 | intergenic | V3 Leaf DM,V3 Total DM |  |
| Zm00001d043622 | uncharacterized LOC103651255 | 3 | NC_024461.2 | 205433975 | 205435543 | chr3.S_205408754 | intergenic | V3 Leaf DM,V6 Sheath DM | Y |
| GRMZM2G004119 | E3 ubiquitin-protein ligase hel2 | 9 | NC_024467.2 | 152408564 | 152412656 | chr9.S_152441427 | intergenic | V3 Sheath DM | Y |
| GRMZM2G032163 | nudix hydrolase 3 | 4 | NC_024462.2 | 177643213 | 177655796 | chr4.S_177658425 | intergenic | V3 Sheath DM | Y |
| GRMZM2G035632 | uncharacterized LOC107403239 | 5 | NC_024463.2 | 15493285 | 15494726 | chr5.S_15550547 | intergenic | V3 Sheath DM |  |
| GRMZM2G041415 | transcription factor MYB8 | 3 | NC_024461.2 | 140875440 | 140876594 | chr3.S_140853075 | intergenic | V3 Sheath DM |  |
| GRMZM2G046587 | Soluble inorganic pyrophosphatase 2 | 5 | NC_024463.2 | 206299750 | 206304035 | chr5.S_206305612 | intergenic | V3 Sheath DM |  |
| GRMZM2G077004 | aspartic proteinase CDR1 | 7 | NC_024465.2 | 167905808 | 167935323 | chr7.S_168006969 | intergenic | V3 Sheath DM | Y |
| GRMZM2G092256 | uncharacterized LOC100276065 | 9 | NC_024467.2 | 86533096 | 86547027 | chr9.S_86299071 | intergenic | V3 Sheath DM |  |
| GRMZM2G092616 | PH, RCC1 and FYVE domains-containing protein 1 | 8 | NC_024466.2 | 161834418 | 161839775 | chr8.S_161901364 | intergenic | V3 Sheath DM | Y |
| GRMZM2G138770 | ATPase 3 | 2 | NC_024460.2 | 36039071 | 36040897 | chr2.S_36084824 | intergenic | V3 Sheath DM | Y |
| GRMZM2G139815 | WRKY74 - superfamily of TFs having WRKY and zinc finger domains | 7 | NC_024465.2 | 95252065 | 95254089 | chr7.S_95029121 | intergenic | V3 Sheath DM |  |
| GRMZM2G147917 | glycerol-3-phosphate 2-O-acyltransferase 6 | 1 | NC_024459.2 | 278510544 | 278512418 | chr1.S_278450781 | intergenic | V3 Sheath DM | Y |
| GRMZM2G152815 | E3 ubiquitin-protein ligase hel2 | 9 | NC_024467.2 | 152499901 | 152504272 | chr9.S_152441427 | intergenic | V3 Sheath DM | Y |
| GRMZM2G164088 | uncharacterized LOC100273027 | 1 | NC_024459.2 | 278405134 | 278408370 | chr1.S_278450781 | intergenic | V3 Sheath DM | Y |
| GRMZM2G332390 | SAUR16 - auxin-responsive SAUR family member | 5 | NC_024463.2 | 15566891 | 15567660 | chr5.S_15550547 | intergenic | V3 Sheath DM |  |
| GRMZM2G347767 | uncharacterized LOC100280127 | 5 | NC_024463.2 | 206342996 | 206346144 | chr5.S_206305612 | intergenic | V3 Sheath DM |  |
| GRMZM2G420310 | Transmembrane 9 superfamily member 1 | 3 | NC_024461.2 | 140623822 | 140629561 | chr3.S_140853075 | intergenic | V3 Sheath DM |  |
| GRMZM5G821252 | peptide transporter PTR2 | 9 | NC_024467.2 | 86006707 | 86015257 | chr9.S_86299071 | intergenic | V3 Sheath DM |  |
| GRMZM5G884544 | uncharacterized LOC100217234 | 8 | NC_024466.2 | 78406100 | 78411079 | chr8.S_78409344 | intronic | V3 Sheath DM | Y |
| GRMZM2G353553 | uncharacterized LOC100193071 | 1 | NC_024459.2 | 83544940 | 83547374 | chr1.S_83566398 | intergenic | V3 Sheath DM,V3 Total DM | Y |
| GRMZM2G003734 | uncharacterized LOC100282554 | 8 | NC_024466.2 | 141824659 | 141849955 | chr8.S_141924122 | intergenic | V3 Total DM |  |
| GRMZM2G016922 | dolabradiene synthase KSL4, chloroplastic | 1 | NC_024459.2 | 240465139 | 240471273 | chr1.S_240486166 | intergenic | V3 Total DM | Y |
| GRMZM2G036765 | Cell division control protein 48 homolog D | 1 | NC_024459.2 | 240510341 | 240515727 | chr1.S_240486166 | intergenic | V3 Total DM | Y |
| GRMZM2G039254 | uncharacterized LOC100381630 | 5 | NC_024463.2 | 76211155 | 76218682 | chr5.S_75935708 | intergenic | V3 Total DM |  |
| GRMZM2G064969 | HXXXD-type acyl-transferase family protein | 4 | NC_024462.2 | 40666790 | 40668352 | chr4.S_40685491 | intergenic | V3 Total DM |  |
| GRMZM2G070515 | uncharacterized LOC103632986 | 7 | NC_024465.2 | 140682189 | 140688136 | chr7.S_140707352 | intergenic | V3 Total DM |  |
| GRMZM2G081653 | uncharacterized LOC100274051 | 8 | NC_024466.2 | 142037333 | 142043518 | chr8.S_141924122 | intergenic | V3 Total DM |  |
| GRMZM2G092483 | RTCN | 1 | NC_024459.2 | 10986942 | 10988129 | chr1.S_10960811 | intergenic | V3 Total DM | Y |
| GRMZM2G093050 | eukaryotic initiation factor 3 | 3 | NC_024461.2 | 6554008 | 6561780 | chr3.S_6432406 | intergenic | V3 Total DM |  |
| GRMZM2G152419 | Membrane metalloprotease ARASP chloroplastic | 1 | NC_024459.2 | 249769850 | 249771485 | chr1.S_249700075 | intergenic | V3 Total DM | Y |
| GRMZM2G158153 | uncharacterized LOC100193964 | 4 | NC_024462.2 | 40796422 | 40801587 | chr4.S_40685491 | intergenic | V3 Total DM |  |
| GRMZM2G165769 | Clavaminate synthase-like protein | 3 | NC_024461.2 | 6376907 | 6379594 | chr3.S_6432406 | intergenic | V3 Total DM |  |
| GRMZM2G167886 | Transducin family protein / WD-40 repeat family protein | 2 | NC_024460.2 | 177986496 | 178020080 | chr2.S_177952190 | intergenic | V3 Total DM |  |
| GRMZM2G312939 | uncharacterized LOC103626560 | 5 | NC_024463.2 | 75635676 | 75639776 | chr5.S_75935708 | intergenic | V3 Total DM |  |
| GRMZM2G329159 | Krueppel-like factor 12 | 1 | NC_024459.2 | 10875393 | 10878343 | chr1.S_10960811 | intergenic | V3 Total DM | Y |
| GRMZM2G412674 | kelch motif family protein | 1 | NC_024459.2 | 14612150 | 14616020 | chr1.S_14624662 | intergenic | V3 Total DM |  |
| GRMZM2G458164 | glucan endo-1,3-beta-glucosidase A6 | 7 | NC_024465.2 | 140751352 | 140754907 | chr7.S_140707352 | intergenic | V3 Total DM |  |
| GRMZM5G838828 | leucine-rich repeat receptor-like protein kinase pseudogene | 1 | NC_024459.2 | 5332032 | 5333375 | chr1.S_5337253 | intergenic | V3 Total DM |  |
| GRMZM5G873586 | uncharacterized LOC100501207 | 10 | NC_024468.2 | 2854312 | 2860169 | chr10.S_2846082 | intergenic | V3 Total DM | Y |
| TIDP3219 | uncharacterized LOC100272871 | 1 | NC_024459.2 | 5411996 | 5413874 | chr1.S_5337253 | intergenic | V3 Total DM |  |
| Zm00001d033075 | uncharacterized LOC103643621 | 1 | NC_024459.2 | 249662816 | 249665754 | chr1.S_249700075 | intergenic | V3 Total DM | Y |
| Zm00001d036651 | F10K1.23 | 6 | NC_024464.2 | 96421942 | 96426359 | chr6.S_96338681 | intergenic | V3 Total DM |  |
| CYP714B3 | uncharacterized LOC100383295 | 1 | NC_024459.2 | 55371829 | 55378346 | chr1.S_55382413 | intergenic | V6 Leaf DM |  |
| GRMZM2G041724 | pre-mRNA-splicing factor 18 | 10 | NC_024468.2 | 132426407 | 132438618 | chr10.S_132394299 | intergenic | V6 Leaf DM |  |
| GRMZM2G045117 | BCL-2 binding anthanogene-1 | 2 | NC_024460.2 | 38333724 | 38335478 | chr2.S_38292371 | intergenic | V6 Leaf DM |  |
| GRMZM2G057766 | chitinase 1 | 5 | NC_024463.2 | 202840780 | 202841842 | chr5.S_202819881 | intergenic | V6 Leaf DM |  |
| GRMZM2G063262 | Steroid nuclear receptor ligand-binding | 2 | NC_024460.2 | 38268761 | 38272882 | chr2.S_38292371 | intergenic | V6 Leaf DM |  |
| GRMZM2G074689 | uncharacterized LOC100274368 | 10 | NC_024468.2 | 129877166 | 129881523 | chr10.S_129918702 | intergenic | V6 Leaf DM | Y |
| GRMZM2G081603 | uncharacterized LOC103627562 | 5 | NC_024463.2 | 202751155 | 202753090 | chr5.S_202819881 | intergenic | V6 Leaf DM |  |
| GRMZM2G088231 | uncharacterized LOC100282153 | 8 | NC_024466.2 | 5938372 | 5944476 | chr8.S_5914760 | intergenic | V6 Leaf DM |  |
| GRMZM2G096090 | uncharacterized LOC103641852 | 10 | NC_024468.2 | 132297643 | 132300315 | chr10.S_132394299 | intergenic | V6 Leaf DM |  |
| GRMZM2G115805 | uncharacterized LOC100273106 | 2 | NC_024460.2 | 90579888 | 90586374 | chr2.S_90259002 | intergenic | V6 Leaf DM |  |
| GRMZM2G154893 | uncharacterized protein DDB_G0271670 | 8 | NC_024466.2 | 5863066 | 5864936 | chr8.S_5914760 | intergenic | V6 Leaf DM |  |
| GRMZM2G158232 | 16.9 kDa class I heat shock protein 1 | 3 | NC_024461.2 | 19966122 | 19966871 | chr3.S_19947370 | intergenic | V6 Leaf DM |  |
| GRMZM2G316721 | uncharacterized LOC100304034 | 10 | NC_024468.2 | 144989745 | 144991292 | chr10.S_144982989 | intergenic | V6 Leaf DM |  |
| GRMZM2G356076 | probable LRR receptor-like serine/threonine-protein kinase At1g74360 | 7 | NC_024465.2 | 168544319 | 168548467 | chr7.S_168577106 | intergenic | V6 Leaf DM |  |
| GRMZM2G373527 | transducin family protein / WD-40 repeat family protein | 7 | NC_024465.2 | 101004710 | 101009817 | chr7.S_101123109 | intergenic | V6 Leaf DM |  |
| GRMZM2G422240 | class I heat shock protein pseudogene | 3 | NC_024461.2 | 19868730 | 19869487 | chr3.S_19947370 | intergenic | V6 Leaf DM |  |
| Zm00001d020228 | uncharacterized LOC100278047 | 7 | NC_024465.2 | 101174397 | 101177296 | chr7.S_101123109 | intergenic | V6 Leaf DM |  |
| Zm00001d029033 | uncharacterized LOC100191594 | 1 | NC_024459.2 | 55450346 | 55453796 | chr1.S_55382413 | intergenic | V6 Leaf DM |  |
| Zm00001d043484 | homeobox-DDT domain protein RLT2 - long chain acyl-CoA synthetase pseudogene | 3 | NC_024461.2 | 201624951 | 201630399 | chr3.S_201626811 | exonic | V6 Leaf DM | Y |
| Zm00001d044362 | glycogen synthase kinase-3 homolog MsK-3 | 3 | NC_024461.2 | 226114990 | 226115603 | chr3.S_226133994 | intergenic | V6 Leaf DM |  |
| GRMZM2G167856 | MEK homolog 1 | 3 | NC_024461.2 | 205101800 | 205107142 | chr3.S_205015321 | intergenic | V6 Leaf DM,V6 Sheath DM,V6 Total DM |  |
| GRMZM2G171373 | hexokinase6 | 3 | NC_024461.2 | 204995530 | 205005570 | chr3.S_205015321 | intergenic | V6 Leaf DM,V6 Sheath DM,V6 Total DM |  |
| GRMZM2G092120 | uncharacterized LOC100281537 | 8 | NC_024466.2 | 173694205 | 173701846 | chr8.S_173702930 | intergenic | V6 Leaf DM,V6 Stalk DM,V6 Total DM | Y |
| CKX10 | cytokinin dehydrogenase 10 | 1 | NC_024459.2 | 210548729 | 210552137 | chr1.S_210668620 | intergenic | V6 Leaf DM,V6 Total DM | Y |
| GRMZM2G035217 | UPF0496 protein 1 | 1 | NC_024459.2 | 23557767 | 23563097 | chr1.S_23564844 | intergenic | V6 Leaf DM,V6 Total DM | Y |
| GRMZM2G042412 | copper transporter 1 | 3 | NC_024461.2 | 196538906 | 196539686 | chr3.S_196528094 | intergenic | V6 Leaf DM,V6 Total DM | Y |
| GRMZM2G045732 | uncharacterized LOC100273147 | 2 | NC_024460.2 | 211134829 | 211142085 | chr2.S_211156672 | intergenic | V6 Leaf DM,V6 Total DM | Y |
| GRMZM2G128644 | putative VQ motif family protein | 1 | NC_024459.2 | 52748869 | 52750058 | chr1.S_52767074 | intergenic | V6 Leaf DM,V6 Total DM | Y |
| GRMZM2G314064 | WUSCHEL-related homeobox 6 | 1 | NC_024459.2 | 53579643 | 53583329 | chr1.S_53621515 | intergenic | V6 Leaf DM,V6 Total DM | Y |
| GRMZM2G407825 | phospholipid-transporting ATPase 1 | 1 | NC_024459.2 | 53748836 | 53756506 | chr1.S_53621515 | intergenic | V6 Leaf DM,V6 Total DM | Y |
| GRMZM6G865522 | guanylate kinase | 1 | NC_024459.2 | 52836664 | 52841240 | chr1.S_52767077 | intergenic | V6 Leaf DM,V6 Total DM | Y |
| pco092737 | uncharacterized LOC100192571 | 2 | NC_024460.2 | 211208651 | 211216294 | chr2.S_211156672 | intergenic | V6 Leaf DM,V6 Total DM | Y |
| Zm00001d002111 | uncharacterized LOC103648361 | 2 | NC_024460.2 | 6049326 | 6051449 | chr2.S_6053391 | intergenic | V6 Leaf DM,V6 Total DM | Y |
| GRMZM2G061537 | calmodulin-binding receptor-like cytoplasmic kinase 3 | 10 | NC_024468.2 | 129941603 | 129946477 | chr10.S_129941704 | UTR5 | V6 Leaf DM;V6 Total DM | Y |
| EMB1586 | uncharacterized LOC100191447 | 5 | NC_024463.2 | 193469647 | 193474061 | chr5.S_193438077 | intergenic | V6 Sheath DM |  |
| GRMZM2G000221 | PVR3-like protein | 7 | NC_024465.2 | 32221939 | 32222607 | chr7.S_32102121 | intergenic | V6 Sheath DM |  |
| GRMZM2G016393 | 40S ribosomal protein S4-C | 5 | NC_024463.2 | 152160477 | 152162101 | chr5.S_151974480 | intergenic | V6 Sheath DM | Y |
| GRMZM2G049318 | dirigent protein 24 | 9 | NC_024467.2 | 141479289 | 141480543 | chr9.S_141479857 | exonic | V6 Sheath DM |  |
| GRMZM2G057408 | uncharacterized LOC100283068 | 6 | NC_024464.2 | 95752341 | 95758204 | chr6.S_95715567 | intergenic | V6 Sheath DM |  |
| GRMZM2G071771 | PVR3-like protein | 7 | NC_024465.2 | 32081025 | 32081651 | chr7.S_32102121 | intergenic | V6 Sheath DM |  |
| GRMZM2G074790 | Chaperonin CPN60-like 2 mitochondrial | 6 | NC_024464.2 | 165907956 | 165913306 | chr6.S_165981165 | intergenic | V6 Sheath DM |  |
| GRMZM2G086707 | uncharacterized LOC103644394 | 1 | NC_024459.2 | 6207098 | 6208131 | chr1.S_6233904 | intergenic | V6 Sheath DM | Y |
| GRMZM2G087850 | phosphatidylinositol synthase 2 | 6 | NC_024464.2 | 95649654 | 95655774 | chr6.S_95715567 | intergenic | V6 Sheath DM |  |
| GRMZM2G088995 | phosphoribosylamine--glycine ligase | 6 | NC_024464.2 | 165987027 | 165991710 | chr6.S_165981165 | intergenic | V6 Sheath DM |  |
| GRMZM2G091245 | uncharacterized LOC100277966 | 4 | NC_024462.2 | 61379820 | 61395297 | chr4.S_61400481 | intergenic | V6 Sheath DM |  |
| GRMZM2G092146 | uncharacterized LOC100275352 | 9 | NC_024467.2 | 99851803 | 99852714 | chr9.S_99849518 | intergenic | V6 Sheath DM |  |
| GRMZM2G115948 | proteasome subunit alpha type-2 | 5 | NC_024463.2 | 193379028 | 193381340 | chr5.S_193438077 | intergenic | V6 Sheath DM |  |
| GRMZM2G116538 | remorin | 10 | NC_024468.2 | 134081682 | 134098975 | chr10.S_134056001 | intergenic | V6 Sheath DM |  |
| GRMZM2G143499 | uncharacterized LOC100283840 | 10 | NC_024468.2 | 146663667 | 146667030 | chr10.S_146653414 | intergenic | V6 Sheath DM |  |
| GRMZM2G148706 | putative RING zinc finger domain superfamily protein | 1 | NC_024459.2 | 19323239 | 19324713 | chr1.S_19328048 | intergenic | V6 Sheath DM | Y |
| GRMZM2G166718 | ATP-dependent DNA helicase chloroplastic | 2 | NC_024460.2 | 231171230 | 231187833 | chr2.S_231146674 | intergenic | V6 Sheath DM | Y |
| GRMZM2G166776 | uncharacterized LOC100274774 | 2 | NC_024460.2 | 231129791 | 231130667 | chr2.S_231146674 | intergenic | V6 Sheath DM | Y |
| GRMZM2G318412 | uncharacterized LOC109944439 | 2 | NC_024460.2 | 233973098 | 233975586 | chr2.S_233740451 | intergenic | V6 Sheath DM |  |
| GRMZM2G448701 | uncharacterized LOC103631379 | 1 | NC_024459.2 | 6237633 | 6239706 | chr1.S_6233904 | intergenic | V6 Sheath DM | Y |
| Zm00001d020588 | eukaryotic translation initiation factor 3 subunit E | 7 | NC_024465.2 | 123766260 | 123767567 | chr7.S_123781663 | intergenic | V6 Sheath DM | Y |
| Zm00001d020589 | uncharacterized LOC109940794 | 7 | NC_024465.2 | 123818191 | 123819107 | chr7.S_123781663 | intergenic | V6 Sheath DM | Y |
| Zm00001d025931 | uncharacterized LOC103641878 | 10 | NC_024468.2 | 134023315 | 134029068 | chr10.S_134056001 | intergenic | V6 Sheath DM |  |
| GRMZM2G002104 | uncharacterized LOC103647482 | 2 | NC_024460.2 | 191770572 | 191772062 | chr2.S_191773591 | intergenic | V6 Stalk DM |  |
| GRMZM2G023392 | expp1 protein | 1 | NC_024459.2 | 254400095 | 254402806 | chr1.S_254402180 | intronic | V6 Stalk DM |  |
| GRMZM2G026892 | uncharacterized LOC100193214 | 5 | NC_024463.2 | 23782105 | 23785628 | chr5.S_23791969 | intergenic | V6 Stalk DM | Y |
| GRMZM2G038281 | uncharacterized LOC100285111 | 3 | NC_024461.2 | 175449757 | 175457087 | chr3.S_175504593 | intergenic | V6 Stalk DM |  |
| GRMZM2G050845 | RAP | 3 | NC_024461.2 | 4838546 | 4841543 | chr3.S_4861726 | intergenic | V6 Stalk DM | Y |
| GRMZM2G056462 | eukaryotic translation initiation factor 2 beta subunit | 1 | NC_024459.2 | 56094245 | 56104042 | chr1.S_56074001 | intergenic | V6 Stalk DM |  |
| GRMZM2G060349 | uncharacterized LOC100382240 | 5 | NC_024463.2 | 23853139 | 23883140 | chr5.S_23791969 | intergenic | V6 Stalk DM | Y |
| GRMZM2G070881 | NEDD8 ultimate buster 1 | 2 | NC_024460.2 | 5873607 | 5878129 | chr2.S_5925885 | intergenic | V6 Stalk DM |  |
| GRMZM2G074037 | 26S proteasome regulatory subunit 10B homolog A | 9 | NC_024467.2 | 94817007 | 94823777 | chr9.S_94793185 | intergenic | V6 Stalk DM |  |
| GRMZM2G084086 | tyrosine specific protein phosphatase family protein | 5 | NC_024463.2 | 216610134 | 216612826 | chr5.S_216619301 | intergenic | V6 Stalk DM |  |
| GRMZM2G084116 | uncharacterized LOC103627800 | 5 | NC_024463.2 | 216620120 | 216623083 | chr5.S_216619301 | intergenic | V6 Stalk DM |  |
| GRMZM2G085509 | ergosterol biosynthetic protein 28 | 3 | NC_024461.2 | 47105144 | 47106125 | chr3.S_47010705 | intergenic | V6 Stalk DM | Y |
| GRMZM2G100288 | Receptor-like protein kinase FERONIA | 1 | NC_024459.2 | 56040048 | 56042979 | chr1.S_56074001 | intergenic | V6 Stalk DM |  |
| GRMZM2G107838 | uncharacterized LOC100280333 | 1 | NC_024459.2 | 44536546 | 44539418 | chr1.S_44539954 | upstream | V6 Stalk DM | Y |
| GRMZM2G160136 | transcriptional activator TAF-1-like | 3 | NC_024461.2 | 47004130 | 47005176 | chr3.S_47010705 | intergenic | V6 Stalk DM | Y |
| GRMZM2G170489 | uncharacterized LOC100278093 | 1 | NC_024459.2 | 295712889 | 295716384 | chr1.S_295724637 | intergenic | V6 Stalk DM |  |
| GRMZM2G173416 | uncharacterized LOC100304380 | 2 | NC_024460.2 | 207465555 | 207484585 | chr2.S_207613241 | intergenic | V6 Stalk DM |  |
| GRMZM2G318689 | ethylene receptor 4 | 4 | NC_024462.2 | 235781418 | 235784227 | chr4.S_235763824 | intergenic | V6 Stalk DM |  |
| GRMZM2G361388 | Sterile alpha motif (SAM) domain-containing protein | 2 | NC_024460.2 | 5947225 | 5948320 | chr2.S_5925885 | intergenic | V6 Stalk DM |  |
| GRMZM2G397759 | pentatricopeptide repeat-containing protein pseudogene | 3 | NC_024461.2 | 4929634 | 4931210 | chr3.S_4861726 | intergenic | V6 Stalk DM | Y |
| Zm00001d042655 | signal recognition particle receptor subunit alpha homolog | 3 | NC_024461.2 | 175563093 | 175566534 | chr3.S_175513354 | intergenic | V6 Stalk DM |  |
| Zm00001d046538 | SEC14-like protein 1 | 9 | NC_024467.2 | 94662146 | 94667635 | chr9.S_94793185 | intergenic | V6 Stalk DM |  |
| Zm00001d053130 | uncharacterized LOC109945934 | 4 | NC_024462.2 | 215060486 | 215061050 | chr4.S_215106444 | intergenic | V6 Stalk DM |  |
| GRMZM2G002555 | uncharacterized LOC100281418 | 4 | NC_024462.2 | 215474307 | 215476398 | chr4.S_215441650 | intergenic | V6 Total DM |  |
| GRMZM2G004957 | homeobox-leucine zipper protein ROC7 | 10 | NC_024468.2 | 84124044 | 84129089 | chr10.S_84102202 | intergenic | V6 Total DM | Y |
| GRMZM2G010649 | uncharacterized LOC100280349 | 9 | NC_024467.2 | 25600292 | 25602332 | chr9.S_25593004 | intergenic | V6 Total DM |  |
| GRMZM2G017654 | uncharacterized LOC100274088 | 2 | NC_024460.2 | 3798145 | 3823895 | chr2.S_3806065 | intronic | V6 Total DM |  |
| GRMZM2G021909 | putative ENTH/ANTH/VHS superfamily protein | 6 | NC_024464.2 | 38761324 | 38763476 | chr6.S_39198599 | intergenic | V6 Total DM |  |
| GRMZM2G057717 | uncharacterized LOC103627565 | 5 | NC_024463.2 | 202991016 | 202992019 | chr5.S_203037677 | intergenic | V6 Total DM |  |
| GRMZM2G086410 | thaumatin-like protein 1b | 6 | NC_024464.2 | 39233989 | 39234821 | chr6.S_39198599 | intergenic | V6 Total DM |  |
| GRMZM2G093731 | uncharacterized LOC100384532 | 7 | NC_024465.2 | 120443760 | 120445576 | chr7.S_120205296 | intergenic | V6 Total DM |  |
| GRMZM2G179985 | protein GPR107 | 8 | NC_024466.2 | 174468807 | 174470604 | chr8.S_174419573 | intergenic | V6 Total DM |  |
| GRMZM2G310453 | uncharacterized LOC103641331 | 10 | NC_024468.2 | 84021630 | 84023460 | chr10.S_84102202 | intergenic | V6 Total DM | Y |
| GRMZM2G335685 | uncharacterized LOC100279084 | 5 | NC_024463.2 | 203048786 | 203049513 | chr5.S_203037677 | intergenic | V6 Total DM |  |
| GRMZM2G336557 | uncharacterized LOC100273942 | 8 | NC_024466.2 | 174416887 | 174418247 | chr8.S_174419573 | intergenic | V6 Total DM |  |
| GRMZM2G383540 | DAG protein | 9 | NC_024467.2 | 25547279 | 25549379 | chr9.S_25593004 | intergenic | V6 Total DM |  |
| Zm00001d044841 | uncharacterized protein At3g28850 | 9 | NC_024467.2 | 4900405 | 4902575 | chr9.S_4843905 | intergenic | V6 Total DM |  |
| Zm00001d046264 | probable leucine-rich repeat receptor-like protein kinase At5g49770 | 9 | NC_024467.2 | 77308535 | 77310494 | chr9.S_77309677 | upstream | V6 Total DM |  |
|  |  |  |  |  |  |  |  |  |  |

1) Y: the corresponding gene was validated by multiple GWAS methods.
